# Silent but Invasive: A Case Report of a Sarcomatoid Bladder Carcinoma in a 29-Year-Old Male Presenting As Vesico-Cutaneous Fistula Without Hematuria

**DOI:** 10.7759/cureus.88261

**Published:** 2025-07-18

**Authors:** Neeraj Agarwal, Rajesh K Kumawat, Sarthak Sharma, Prashant Gupta, Dharmendra K Jangid

**Affiliations:** 1 Urology and Renal Transplant Surgery, Sawai Man Singh Medical College, Jaipur, IND

**Keywords:** cutaneous ureterostomy, radical cystectomy, radical cystoprostatectomy, sarcomatoid urothelial carcinoma, suc, tcc, vesicocutaneous fistula

## Abstract

Sarcomatoid urothelial carcinoma (SUC) is a rare and aggressive variant of urinary bladder cancer and is associated with a poor prognosis. This case report presents the unusual case of a 29-year-old male who presented with a vesicocutaneous fistula causing continuous suprapubic urine leakage for four months following a previous bladder surgery for a vesical calculus. This patient had no history of hematuria, which is a common symptom of bladder cancer. Investigations, including contrast-enhanced computed tomography (CECT) and 18F-fluorodeoxyglucose (FDG) positron emission tomography-computed tomography (PET-CT), revealed a large, locally advanced bladder mass infiltrating the anterior abdominal wall with a vesicocutaneous fistula. The patient underwent a radical cystoprostatectomy, wide local excision of the fistulous tract, bilateral pelvic lymph node dissection, right cutaneous ureterostomy, and abdominal wall reconstruction with a pedicled anterolateral thigh flap. Histopathology confirmed poorly differentiated SUC. This case is unique due to the patient's young age, the atypical presentation with a vesicocutaneous fistula without hematuria, and the sarcomatoid histology. It highlights the aggressive nature of SUC, its potential for unusual presentations that can delay diagnosis, and the necessity of considering bladder cancer in young patients with urinary fistulas, even without hematuria. Radical surgery with meticulous reconstruction offers the best chance for disease control in such complex scenarios, emphasizing the importance of early suspicion and multidisciplinary management.

## Introduction

Sarcomatoid carcinoma of the urinary bladder represents a rare variant of urothelial carcinoma (UC), constituting less than 1% of all bladder cancers. This condition is distinguished by a biphasic histology, which comprises both epithelial (urothelial) and mesenchymal (sarcoma-like) components. It is recognized for its aggressive clinical behavior and unfavorable prognosis [1].

The majority of patients exhibit hematuria and irritative voiding symptoms. The occurrence of a vesico-cutaneous fistula is uncommon and, to our knowledge, has not been documented in young individuals with sarcomatoid histology. We present a unique case involving a 29-year-old male diagnosed with sarcomatoid urothelial carcinoma, who exhibited persistent suprapubic urine leakage resulting from a fistula that developed following previous bladder surgery.

## Case presentation

A 29-year-old male laborer presented with continuous leakage of urine from a suprapubic operative scar for four months. He underwent open suprapubic cystotomy with bladder growth biopsy found incidentally during exploration for vesical calculus, which was done outside of our institute five months ago. After Foley's catheter removal three weeks later, he developed a urine leak from the operative scar (five to six diapers/day) and no per urethral voiding of urine since then. He had no history of hematuria, known comorbidities, or any other surgical history. He was a chronic tobacco chewer but had no history of smoking. 

**Figure 1:**
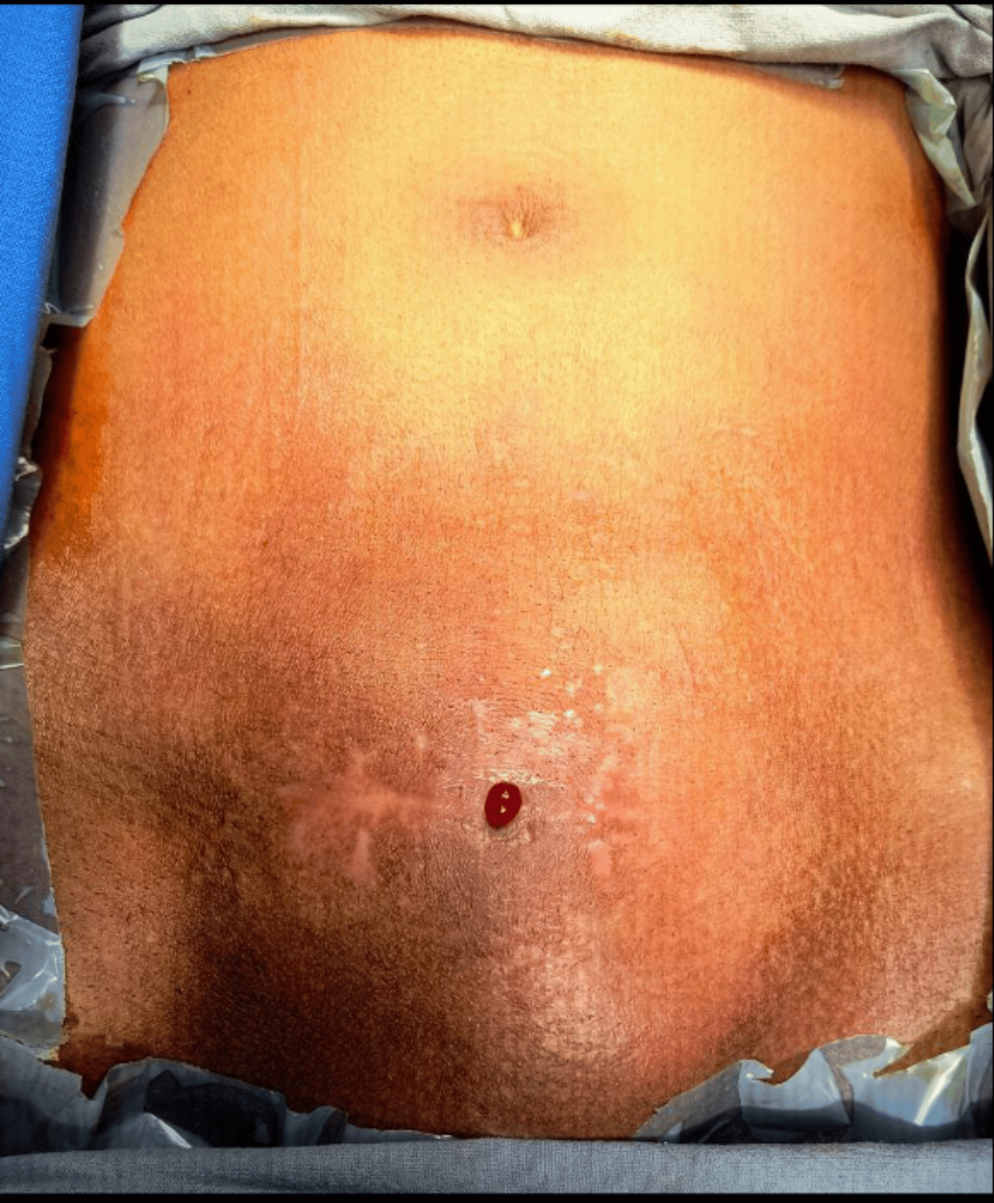
Vesico-cutaneous fistula

On physical examination, the patient was average built with an Eastern Cooperative Oncology Group (ECOG) 2 performance status, and a suprapubic bulge was observed with a ~7cm Pfannenstiel scar one finger breadth above the pubic symphysis (Figure 1).

**Figure 2:**
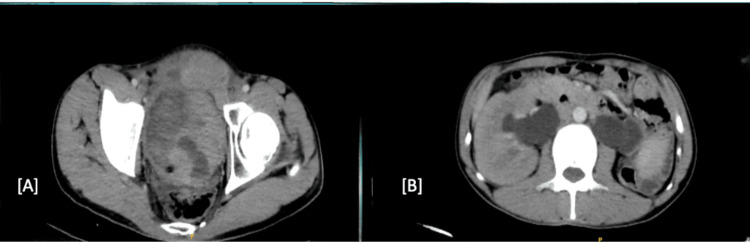
Axial section of contrast-enhanced computed tomography whole abdomen (A) A large heterogeneously enhancing mass lesion in the urinary bladder, which is infiltrating the anterior abdominal wall; (B) Both kidneys show hydroureteronephrosis with a small left kidney.

A midline fistulous opening with continuous urine dribbling was noted. An irregular fixed mass was palpable, spanning ~ 4 cm cranial to the upper border of the pubic symphysis and ~ 10 cm transversely. A digital rectal examination revealed a firm, flat prostate and a large, irregular mass palpable bimanually. We investigated with a contrast-enhanced computed tomography (CECT) of the abdomen and pelvis, which revealed an 89 x 78 mm mass along the posterior basal and lateral wall of the bladder with intra luminal extension, infiltrating the peri vesical space and anterior abdominal wall and had bilateral vesico-ureteric junction (VUJ) involvement with proximal hydroureteronephrosis, left small contracted kidney (58 x 28 mm) and a vesico-cutaneous fistula measuring 34 × 3.1 mm was noted (Figure 2).

**Figure 3:**
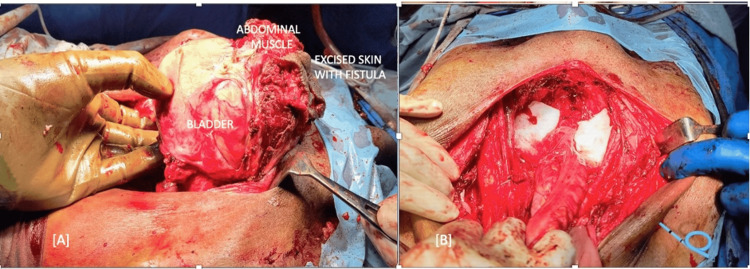
(A) Excision of tumor with involved abdominal skin and rectus abdomens; (B) post resection image with right ureterostomy in situ

He was further evaluated with an 18F-fluorodeoxyglucose (FDG) positron emission tomography-computed tomography (PET-CT) scan for metastatic evaluation, which showed an FDG-avid mass (standardized uptake value (SUV) max 28) involving the bladder base and lateral wall with no distant metastasis. The patient underwent radical cysto-prostectomy with wide local excision of the fistulous tract and bilateral pelvic lymph node dissection with Right cutaneous ureterostomy and ligation of the left ureter because of a small atrophic kidney (Figure 3).

**Figure 4:**
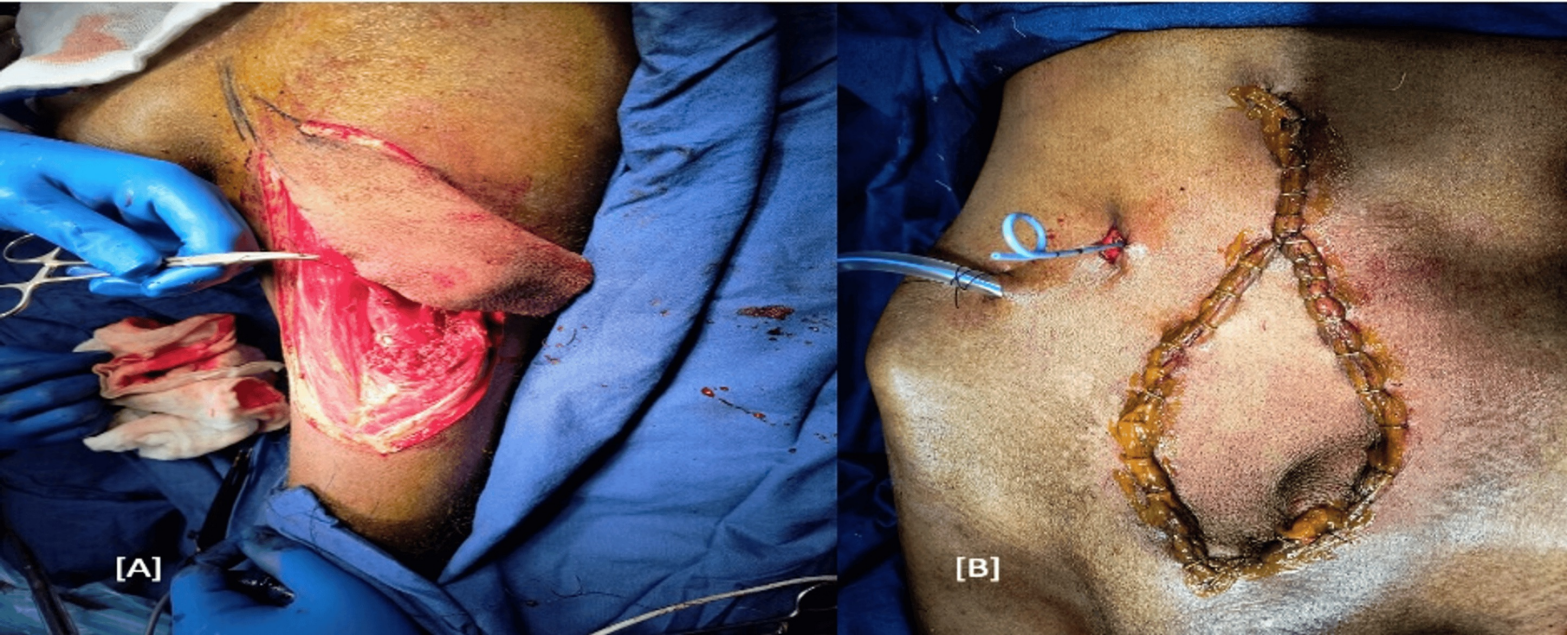
(A) anterolateral thigh flap from left thigh; (B) final operative scar

Reconstruction of a 15 × 8 cm abdominal wall defect was done using a pedicled left anterolateral thigh (ALT) by the plastic surgery department (Figure 4).

**Figure 5:**
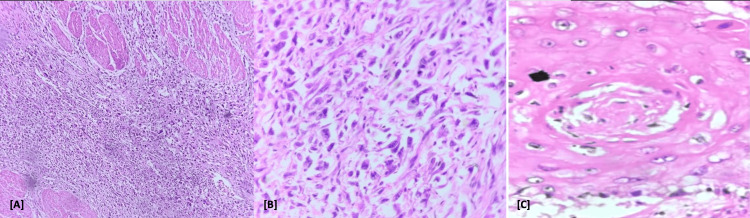
H&E stained smear (A) Low-power (10x) view showing invasive tumor composed of both epithelial and sarcomatoid areas. The tumor is infiltrating the muscularis propria; (B) High-power (40x) view showing spindle-shaped pleomorphic tumor cells with high nuclear atypia and brisk mitotic activity, consistent with sarcomatoid differentiation; (C) Foci of squamous cell carcinoma

Postoperative recovery was uneventful. Right ureterostomy was functional since postoperative day (POD) zero with good output ~1.5 - 2L/day; the patient was allowed orally on POD two and ambulated on POD seven. The patient was discharged in satisfactory condition on POD 14. His histopathology report showed poorly differentiated carcinoma of squamous differentiation, suggesting sarcomatoid urothelial carcinoma (Figure 5).

All regional lymph nodes were negative for metastasis (0/17); muscle propria were invaded by the tumor, and the tumor invaded skeletal muscle and soft tissue with pathological staging pT4bN0.

## Discussion

Bladder cancer is the seventh most prevalent malignancy in men globally. Pure urothelial histology is identified in 75% of cases, whereas non-urothelial histology is present in 15% to 25% of instances. Non-urothelial histology may occur singularly or in combination and encompasses squamous, glandular, small-cell, micropapillary urothelial cancer, nested, microcytic, microtubular, sarcomatoid, high-grade neuroendocrine, and even trophoblastic variants [2].

Sarcomatoid urothelial carcinoma represents a rare variant characterized by aggressive clinical behavior and poor survival outcomes [1,3]. The typical presentation of carcinoma of the urinary bladder is hematuria. Around 20% of bladder cancers present without hematuria, often with irritative voiding symptoms like frequency, urgency, and dysuria. It is usually associated with invasive bladder malignancy or carcinoma in situ [4]. In our case, hematuria was absent despite the high grade and advanced local invasion. The formation of a fistula is rare in bladder cancer and typically indicates aggressive local invasion. The sarcomatoid component may facilitate rapid soft tissue infiltration [5]. In this instance, the patient's young age, absence of per-urethral voiding, and presentation with a vesicocutaneous fistula render this a highly unusual and complex case. Only a limited number of reports have documented bladder cancer presenting with fistula formation (e.g., vesicovaginal or enterovesical) [6]. However, none specifically address sarcomatoid histology with vesicocutaneous involvement in a young adult.

Locally advanced urinary bladder cancer that invades beyond the muscle layer is usually managed through radical cystectomy or neoadjuvant chemotherapy with radical cystectomy. The sarcomatoid variant is known to have a limited response to chemotherapy, and there has been no significant difference in survival on neoadjuvant chemotherapy followed by radical cystectomy or directly with radical cystectomy [3,5,6]. In our case, aggressive surgical management, including wide local excision and abdominal wall reconstruction, was necessitated due to tumor invasion. Radical resection with negative margins is critical for successful outcomes. The anterolateral thigh (ALT) flap provided a durable reconstruction for the abdominal wall. Despite aggressive management, SUC patients have a median overall survival (OS) of just 17.5 months [4].

## Conclusions

Sarcomatoid carcinoma of the urinary bladder represents a rare and aggressive malignancy. This particular case, characterized by the development of a vesicocutaneous fistula in a young adult male, emphasizes the critical need for early diagnostic suspicion and comprehensive management strategies. The implementation of radical surgical intervention, coupled with meticulous reconstruction techniques, offers the optimal opportunity for disease control in such intricate cases. This instance further demonstrates that sarcomatoid carcinoma can manifest in the absence of hematuria, potentially hindering timely diagnosis. Clinicians should maintain a high index of suspicion for bladder cancer in young patients who present with urinary fistulas or obstructive urinary symptoms, even in the absence of hematuria. A multidisciplinary approach to management, which includes thorough histopathological evaluation and radical surgical treatment, remains essential in achieving favorable outcomes.
